# Does gender influence the outcomes of total ankle arthroplasty in patients with ankle osteoarthritis?

**DOI:** 10.1186/s13018-020-01731-5

**Published:** 2020-06-05

**Authors:** Gun-Woo Lee, Keun-Bae Lee

**Affiliations:** Department of Orthopedic Surgery, Chonnam National University Medical School and Hospital, 42 Jebongro, Donggu, Gwangju, 61469 Republic of Korea

**Keywords:** Total ankle arthroplasty, Gender, Ankle osteoarthritis, Clinical outcomes, Survivorship

## Abstract

**Background:**

Total ankle arthroplasty has progressed as a treatment option for patients with ankle osteoarthritis. However, no studies have been conducted to evaluate the effect of gender on the outcome. The purpose of the present study was to evaluate outcomes, survivorship, and complications rates of total ankle arthroplasty, according to gender differences.

**Methods:**

This study included 187 patients (195 ankles) that underwent mobile-bearing HINTEGRA prosthesis at a mean follow-up of 7.5 years (range, 4 to 14). The two groups consisted of a men’s group (106 patients, 109 ankles) and a women’s group (81 patients, 86 ankles). Average age was 64.4 years (range, 45 to 83).

**Results:**

Clinical scores on the Ankle Osteoarthritis Scale for pain and disability, and American Orthopaedic Foot and Ankle Society ankle-hindfoot score improved and the difference was not statistically significant between the two groups at the final follow-up. There were no significant differences in complication rates and implant survivorship between the two groups. The overall survival rate was 96.4% in men and 93.4% in women at a mean follow-up of 7.5 years (*p* = 0.621).

**Conclusions:**

Clinical outcomes, complication rates, and survivorship of total ankle arthroplasty were comparable between men and women. These results suggest that gender did not seem to affect outcomes of total ankle arthroplasty in patients with ankle osteoarthritis.

**Level of evidence:**

Therapeutic level III

## Background

Ankle osteoarthritis is a slowly progressive destructive disease inducing pain, dysfunction, impaired mobility, and decreased quality of life [1]. Compared to ankle arthrodesis, total ankle arthroplasty (TAA) can preserve gait motion by restoring closer to normal joint motion and improve patient satisfaction by reducing pain while minimizing subtalar joint arthritis [2–8]. Several recent studies reported satisfactory outcomes of TAA, these promising outcomes have led TAA as a primary treatment option for ankle osteoarthritis [9–20].

Various predisposing factors including age, gender, and preoperative deformity can affect the outcome of TAA [10, 12, 18, 21–24]. In relation to gender difference, several studies demonstrated divergent clinical outcomes following total hip or total knee arthroplasty. In total hip arthroplasty, some studies reported no difference in all postoperative outcome measures between men and women [25]. On the other hand, another study reported that gender-related analysis resulted in significantly higher scores for men compared to women [26]. Recent meta-analysis demonstrated that men showed a higher risk of revision after primary total hip arthroplasty due to any cause, infection, or aseptic loosening, when compared to women [27]. In terms of total knee arthroplasty, several studies reported that functional outcomes were different between men and women [28–30]. On the contrary, another study suggested that gender does not seem to affect clinical outcome [31].

However, no study has been conducted examining the outcomes of gender-based differences in TAA. We hypothesize that clinical and radiographic outcomes for TAA would be comparable between men and women patients. Thus, the purpose of the present study was to compare intermediate-term clinical and radiographic outcomes, survivorship, and complications rates of TAA in patients with end-stage ankle osteoarthritis according to gender differences.

## Materials and methods

This study was approved by the Institutional Review Board of our hospital. Informed consent was obtained from all patients. Between January 2005 and December 2015, a total of 243 consecutive primary TAA were performed in 231 patients using cementless three-component HINTEGRA prosthesis (Newdeal, Lyon, France/Integra Lifesciences, Plainsboro, NJ, USA). Inclusion criteria were symptomatic end-stage ankle osteoarthritis with a minimum follow-up of 48 months after TAA, and fulfillment of general total ankle arthroplasty indications including good bone stock and normal neurovascular status. Patients were excluded from the trial if they had any of the following: secondary osteoarthritis due to osteonecrosis of the talus or pigmented villonodular synovitis; arthritis caused by systemic disease, such as rheumatoid arthritis and hemophilia; and previous history of infectious arthritis or ankle arthrodesis.

Finally, we enrolled 187 patients (195 ankles) who underwent primary TAA. We divided the patients into two groups according to gender: (1) men’s group (106 patients, 109 ankles) and (2) women’s group (81 patients, 86 ankles). Patients in each group showed similar characteristics in mean age, preoperative diagnosis, and follow-up duration (Table 1).

**Table 1:** Comparison of patient demographics between men and women

	Men (*N* = 109 ankles)	Women (*N* = 86 ankles)	*p* value*
**Age (year)**†	65.4 ± 7.3 (45 to 82)	63.2 ± 7.2 (47 to 83)	0.114
**BMI (kg/m**^**2**^**)**†	25.3 ± 2.3 (20.5 to 31.8)	25.6 ± 2.7 (19.1 to 32.0)	0.111
**Diagnosis**‡			0.277
Primary osteoarthritis	40 (36.7%)	31 (36.0%)	
Recurrent ankle sprain	35 (32.1%)	20 (23.3%)	
Post-fracture	34 (31.2%)	35 (40.7%)	
**Follow-up (months)**†	90.3 ± 32.6 (48 to 170)	89.6 ± 32.9 (50 to 174)	0.887

*BMI* body mass index

*The independent *t* test was used to analyze age differences in age, BMI, and follow-up duration. The chi-square test was used to analyze differences in preoperative diagnoses. A *p* value of < 0.05 was considered significant

†The values are given as the mean ± standard deviation, with the range in parentheses

‡The values are given as the number of ankles, with the percentage in parentheses

### Surgical technique and postoperative management

Patients underwent TAA with HINTEGRA prosthesis by a single surgeon, with use of a longitudinal anterior approach between the anterior tibial tendon and the extensor hallucis longus, with the patient in the supine position. After removal of anterior capsular synovial tissue and osteophytes, the tibial cut was performed perpendicular to the mechanical axis of the tibia. The talus bone cut was made parallel to the tibial cut; then, the medial, lateral, and posterior talar cuts were made. After selected components had been inserted, the alignment, stability, joint motion, and implant position were checked by an image intensifier. Hindfoot alignment and ligamentous stability were rechecked. Concomitant procedures were done to obtain neutral alignment, stability, and ligamentous balance, if necessary.

Postoperatively, for the first postoperative 2 weeks, a short leg splint was worn and patients maintained non-weight-bearing with neutral ankle positions. For the next 4 weeks, patients were allowed to begin range of motion (ROM) exercises. At 6 weeks after surgery, progressive weight-bearing ambulation was initiated with ankle-foot orthosis. The rehabilitation protocol differed slightly if additional procedures were performed at the index surgery.

### Clinical evaluation

Ankle Osteoarthritis Scale (AOS) pain and disability [32], American Orthopaedic Foot and Ankle Society (AOFAS) ankle-hindfoot scoring system [33], Short Form (SF)-36 Physical Component Summary score (PCS) [34], and visual analog scale (VAS) for pain were administered to the patients for assessment of clinical outcomes. ROM of ankle joint, including degree of dorsiflexion and plantar flexion was measured using a goniometer along the lateral border of the leg and foot. The main outcomes were assessed based on the AOS, because it was reliable, validated, and an ankle joint-specific measurement, and it is clinically important to evaluate whether the AOS improved beyond the minimal clinically important difference (MCID). The MCID is the smallest change in outcome assessment in patient who identified themselves as minimally better or worse after surgical procedure [35]. The AOFAS scoring system, a combination of subjective and objective data, was used as secondary assessment tool. SF-36 scores and VAS pain were utilized as comparative measures.

This study noted postoperative complications, and these were divided into major and minor. Major complications included deep infection, periprosthetic osteolysis, and its associated complication. Minor complications included wound problems and heterotopic ossification [36, 37]. We defined failure when either the tibial or talar metallic component was replaced [22].

### Radiographic evaluation

Radiographic examination included anteroposterior and lateral radiographs of the ankle on full weight-bearing, taken preoperatively and postoperatively. Follow-up radiographs were taken at 1, 3, 6, and 12 months postoperatively, and annually thereafter. The coronal plane alignment was determined from the angle formed between the anatomical axis of the tibia and a line perpendicular to the talus or talar component. For the radiographic analysis, measurements were made using a PACS (picture archiving and communication system: Maroview 5.4; INFINITT Healthcare). To minimize measurement errors, by two independent observers measured radiographic values twice and the mean of the remaining values except the maximum and the minimum was reported.

### Statistical analysis

Descriptive statistics were calculated by use of standard formulas. A Kolmogorov-Smirnov test was used to verify that data were normally distributed. For normally distributed variables, the independent *t* test was used to determine the significance of intergroup differences. The Mann-Whitney *U* test was used to analyze differences when there was a lack of normality. For the categorical variables, chi-square test or Fisher’s exact test was used to analyze differences. Data were analyzed using SPSS version 23.0 (IBM Corp., Armonk, NY, USA). Statistical significance was accepted for *p* values < 0.05, and all components of the statistical analysis were reviewed by a statistician.

## Results

### Clinical outcomes

**Fig. 1:**
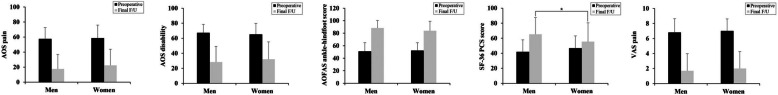
The mean preoperative and final follow-up clinical values are shown as bar graphs and the gap between the two values is the improvement value. The error bars indicate standard deviation. AOS, Ankle Osteoarthritis Scale; AOFAS, American Orthopaedic Foot and Ankle Society; SF-36 PCS, Short Form-36 Physical Component Summary; VAS, visual analog scale. *Significant differences in SF-36 PCS scores between the two groups at the final follow-up

The clinical outcome details are provided in Table 2 (Fig. 1). The mean preoperative AOS pain scores were 57.6 points in men and 58.6 points in women, and these were improved to 17.6 and 22.4 points, respectively, at final follow-up. The mean AOS disability scores were 67.3 points in men and 65.2 points in women, and these were improved to 28.3 and 32.1 points, respectively, at final follow-up. According to a previous study [35], the MCID of the total AOS (mean of AOS pain and disability) was 28.0 points. In the present study, the mean improvement of the total AOS was 39.5 points in men and 34.7 points in women. Therefore, the two groups showed clinically meaningful improvement after TAA. The preoperative AOFAS scores were improved to 88.3 points in men and 83.9 points in women at final follow-up. The SF-36 PCS was improved by 23.2 points in men and 8.8 points in women. The VAS pain score was also improved to 1.7 points in men and 2.0 points in women at final follow-up. There was no significant difference in clinical outcomes and ROM between the two groups at the final follow-up, except for SF-36 PCS (*p* < 0.05).

**Table 2:** Clinical outcomes of the men and women groups

	Men (*N* = 109 ankles)	Women (*N* = 86 ankles)	*p* value*
**AOS pain**†			
Preoperative	57.6 ± 14.95	58.6 ± 17.36	0.907
Final follow-up	17.6 ± 19.29	22.4 ± 21.37	0.178
**AOS disability**†			
Preoperative	67.3 ± 11.29	65.2 ± 14.63	0.669
Final follow-up	28.3 ± 20.88	32.1 ± 23.05	0.366
**AOFAS ankle-hindfoot score**†			
Preoperative	51.0 ± 13.92	52.3 ± 12.54	0.573
Final follow-up	88.3 ± 11.99	83.9 ± 15.16	0.060
**SF-36 PCS score**†			
Preoperative	41.9 ± 15.92	46.7 ± 16.45	0.149
Final follow-up	65.2 ± 22.32	55.4 ± 25.05	0.027
**VAS pain**†			
Preoperative	6.8 ± 1.83	7.0 ± 1.61	0.534
Final follow-up	1.7 ± 2.28	2.0 ± 2.25	0.272
**ROM, dorsiflexion (°)**†			
Preoperative	9.8 ± 6.20	9.6 ± 5.44	0.877
Final follow-up	10.5 ± 6.24	11.3 ± 5.12	0.381
**ROM, plantar flexion (°)**†			
Preoperative	21.4 ± 10.09	23.9 ± 9.94	0.138
Final follow-up	22.8 ± 11.28	25.6 ± 9.84	0.170

*AOS* Ankle Osteoarthritis Scale, *AOFAS* American Orthopaedic Foot & Ankle Society, *SF-36 PCS* Short Form-36 Physical Component Summary, *VAS* visual analog scale, *ROM* range of motion

*The independent *t* test was used to analyze age differences in all variables except range of motion. The Mann-Whitney *U* test was used to analyze differences in range of motion. A *p* value of < 0.05 was considered significant

†The values are given as the mean ± standard deviation

### Radiographic outcomes

The radiographic outcome details are provided in Table 3. The mean tibiotalar angle of each group was 8.5° and 11.0° preoperatively, and 3.6° and 4.0° at the final follow-up in men and women, respectively. Significant differences in the preoperative tibiotalar angle and improvement were observed between men and women (preoperative, *p* = 0.020; improvement, *p* = 0.014). However, there was no significant difference in the final follow-up tibiotalar angle (*p* = 0.226).

**Table 3:** Radiographic outcomes of men and women groups

	Men (*N* = 109 ankles)	Women (*N* = 86 ankles)	*p* value*
**Tibiotalar angle (°)**†			
Preoperative	8.5 ± 6.6 (− 31.1 to 19.8)	11.0 ± 6.8 (− 32.0 to 18.4)	0.020
Final follow-up	3.6 ± 2.1 (− 10.2 to 3.2)	4.0 ± 2.2 (− 10.4 to 3.5)	0.226
Improvement‡	4.9 ± 6.3 (− 4.9 to 24.8)	7.5 ± 6.5 (− 4.2 to 26.8)	0.014

*The independent *t* test was used to analyze age differences in tibiotalar angle. A *p* value of < 0.05 was considered significant

†The values are given as the mean ± standard deviation, with the range in parentheses. To reduce errors in the mean calculation due to positive (valgus) and negative (varus) values, these values are calculated as absolute values

‡The improvement is given as the mean difference (preoperative minus final)

### Complications and survivorship

**Fig. 2:**
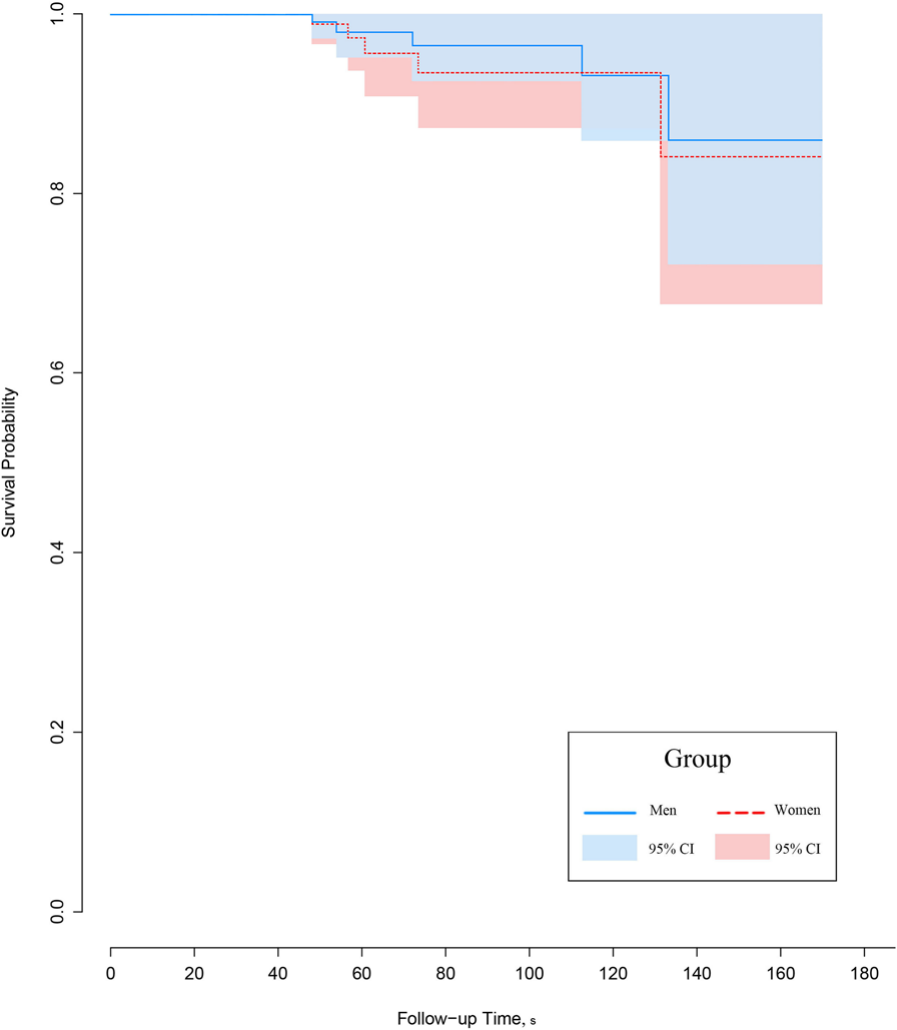
The Kaplan-Meier curve demonstrates the survivorship of total ankle arthroplasty (195 ankles), when the end point was defined as failure of the prosthesis. The overall survival probability of the implant was 96.1% (96.4% in the men’s group and 93.4% in the women’s group) at the mean follow-up of 7.5 years (*p* = 0.621)

There were 19 ankles with complications in men, 20 ankles in women (Table 4). The overall survivorship rate was 96.1%, at a mean follow-up of 7.5 years. There was no significant difference between the two groups (*p* = 0.621; log-rank test). Survivorship using Kaplan-Meier curves is shown in Fig. 2.

**Table 4:** Rate of complications associated with total ankle arthroplasty

	Men (*N* = 109 ankles)	Women (*N* = 86 ankles)	*p* value*
**Major complications**†	11 (10.1%)	16 (18.6%)	0.087
Deep infection	1 (0.9%)‡	-	1.000
Periprosthetic osteolysis	10 (9.2%)	16 (18.6%)	0.054
Alone	6 (5.5%)	11 (12.8%)	0.073
With aseptic loosening	-	4 (4.7%)‡	0.036
With component subsidence	4 (3.7%)‡	1 (1.2%)‡	0.386
**Minor complication**†	8 (7.3%)	4 (4.7%)	0.109
Wound problem	-	1 (1.2%)	0.441
Heterotopic ossification	8 (7.3%)	3 (3.5%)	0.352
**Total (no. [%])**	19 (17.4%)	20 (23.3%)	0.459

*The chi-square or Fisher’s exact test was used to analyze differences in the number of complication. A *p* value of < 0.05 was considered significant

†The values are given as the number of ankles, with the percentage in parentheses

‡These are failure cases that were revised with an exchange of the metallic component

There were 10 cases (5.1%) of failure that required exchange of a metallic component, due to deep infection (1 case), osteolysis with aseptic loosening (4 cases), and implant subsidence (5 cases). In the failures, 10 ankles were revised to another TAA or arthrodesis. Seventeen patients with periprosthetic osteolysis alone without loosening or subsidence underwent revision with curettage of an osteolytic lesion, bone grafting, and polyethylene exchange. In those cases, no further progression of osteolytic lesion was reported. Although statistically not significant, women showed higher prevalence of osteolysis and its associated complications compared with men.

One patient had wound problems; however, no one progressed to joint infection, and all healed without further surgical procedures. Three of eleven heterotopic ossification patients underwent resection of ossification due to severe pain.

## Discussion

The most important aim of TAA is to provide an improved and pain-relieved ankle joint. Differences of operative outcomes for TAA between men and women could be expected, since there are differences in bone mineral density (BMD), the prevalence of generalized joint laxity, physical activity, and anatomic variations including alignment of lower extremity and bone size between the genders [27, 38–42]. Thus, the primary purpose of this study was to compare the intermediate-term outcomes of TAA depending on gender difference. In this study, primary outcomes were greatly improved in both groups, but we did not find differences with most patient-reported outcomes, complication rates, and survivorship between the two groups. Consequently, those differences between men and women would not be considered as an affecting factor in the clinical outcomes.

Interestingly, unlike other clinical scores, SF-36 is more likely to have a higher score in men than women. Several studies have demonstrated outcome comparisons between men and women for SF-36 scores of the hip and knee [26, 28]. Cherian et al. analyzed 272 consecutive total knee arthroplasty and reported that the SF-36 PCS was significantly better in men at 7 years of follow-up [28]. Khashan et al. suggested that gender-related analysis resulted in significantly higher scores for men compared to women in SF-36 scores for hip osteoarthritis patients [26]. In regard to knee or hip, men have been reported to score significantly better in the SF-36 test. The activities of daily life have been extensively examined and some studies have reported that men were more tolerant of functional recovery and pain relief, while women on the other hand, tended to perceive their function and pain as more restrictive than they actually were [25, 43].

In terms of radiographic data, the preoperative tibiotalar angles were worse for women than men (Table 3). Since there was a significant difference with tibiotalar angles between men and women before surgery, this was considered to be significantly improved by the process of intra-operative correction. Lee et al. reported that, regardless of preoperative deformity type or degree, if the tibiotalar angle of the ankle was corrected with any ligament imbalance or hindfoot deformity through an additional procedure, the TAA showed good outcomes [22].

The survivorship rate was not significantly different between men and women at a mean follow-up of 7.5 years (96.4% and 93.4%, respectively) (*p* = 0.621) (Fig. 2). Gender had minimal effect on survival rates in TAA, perhaps attributable to the fact that there were no significant differences between the mean age at surgery (*p* = 0.114) or failure rates (*p* = 0.752) between men and women.

In the present study, prevalence of osteolysis and its associated complication was higher in women (16 cases, 18.6%) than in men (10 cases, 9.2%) (Table 4). Men and women are known to have a difference in BMD due to multiple factors including hormonal differences, and we thought these differences may be associated with development of osteolysis. Morakis et al. reported that aseptic loosening was associated with a significant decrease of cortical bone and trabecular bone volumetric-BMD [44]. Runolfsdottir et al. suggested that women had lower BMD than men, and a greater increase in BMD reductions with decreasing female hormones [45]. In other words, the BMD difference between men and women suggests that women are more likely to develop aseptic loosening than men. However, gender was not a risk factor or prognostic factor for revision by aseptic loosening in total knee arthroplasty [46]. Therefore, further studies on the relationship between the prevalence of osteolysis with aseptic loosening and bone mineral density are needed.

There were several weaknesses of our study. First, the present study was conducted retrospectively and the sample size of the two groups was relatively small. This may mitigate the ability to evaluate the influence of baseline characteristics on the outcomes and to detect a difference on this general subject. Second, the overall mean follow-up duration was 7.5 years. This might not be long enough to include late complications. Third, we performed computed tomography (CT) in some cases, but not all cases. Although plain radiograph is the basic assessment for osteolysis, some lesions may be missed or not detected. CT scan can improve the accuracy of the assessment of the development of osteolysis. Finally, this study did not further evaluate objective indicators such as BMD, generalized joint laxity, and whole lower extremity alignment.

## Conclusions

In the intermediate-term follow-up, mobile-bearing total ankle arthroplasty showed similarly good clinical and radiographic outcomes in patients with ankle osteoarthritis, regardless of gender differences. These results suggest that gender did not seem affect outcomes of total ankle arthroplasty. Further studies with larger number of sample sizes and long-term follow-up are needed to clarify the effect of gender on outcomes.

## Data Availability

The datasets analyzed during the current study are not publicly available due to patient confidentiality.

## Abbreviations

TAA: Total ankle arthroplasty
AOS: Ankle Osteoarthritis Scale
AOFAS: American Orthopaedic Foot and Ankle Society
SF-36 PCS: Short Form-36 Physical Component Summary
VAS: Visual analog scale
ROM: Range of motion
BMD: Bone mineral density

## References

[1] Hayes BJ, Gonzalez T, Smith JT, Chiodo CP, Bluman EM (2016). Ankle arthritis: you can't always replace it. J Am Acad Orthop Surg..

[2] Saltzman CL, Kadoko RG, Suh JS (2010). Treatment of isolated ankle osteoarthritis with arthrodesis or the total ankle replacement: a comparison of early outcomes. Clin Orthop Surg..

[3] Pedowitz DI, Kane JM, Smith GM, Saffel HL, Comer C, Raikin SM (2016). Total ankle arthroplasty versus ankle arthrodesis: a comparative analysis of arc of movement and functional outcomes. Bone Joint J..

[4] SooHoo NF, Zingmond DS, Ko CY (2007). Comparison of reoperation rates following ankle arthrodesis and total ankle arthroplasty. J Bone Joint Surg Am..

[5] Dekker TJ, Walton D, Vinson EN (2017). Hindfoot arthritis progression and arthrodesis risk after total ankle replacement. Foot Ankle Int..

[6] Singer S, Klejman S, Pinsker E, Houck J, Daniels T (2013). Ankle arthroplasty and ankle arthrodesis: gait analysis compared with normal controls. J Bone Joint Surg Am.

[7] Lawton CD, Butler BA, Dekker RG, Prescott A, Kadakia AR (2017). Total ankle arthroplasty versus ankle arthrodesis-a comparison of outcomes over the last decade. J Orthop Surg Res..

[8] Gougoulias NE, Khanna A, Maffulli N (2009). History and evolution in total ankle arthroplasty. Br Med Bull..

[9] Barg A, Elsner A, Anderson AE, Hintermann B (2011). The effect of three-component total ankle replacement malalignment on clinical outcome: pain relief and functional outcome in 317 consecutive patients. J Bone Joint Surg Am..

[10] Lee GW, Seon JK, Kim NS, Lee KB (2019). Comparison of intermediate-term outcomes of total ankle arthroplasty in patients younger and older than 55 years. Foot Ankle Int..

[11] Lee GW, Santoso A, Lee KB (2019). Comparison of intermediate-term outcomes of total ankle arthroplasty in primary and ligamentous post-traumatic osteoarthritis. Foot Ankle Int..

[12] Lee GW, Lee KB (2019). Outcomes of total ankle arthroplasty in ankles with >20° of coronal plane deformity. J Bone Joint Surg Am..

[13] Nunley JA, Caputo AM, Easley ME, Cook C (2012). Intermediate to long-term outcomes of the STAR total ankle replacement: the patient perspective. J Bone Joint Surg Am..

[14] Barg A, Zwicky L, Knupp M, Henninger HB, Hintermann B (2013). HINTEGRA total ankle replacement: survivorship analysis in 684 patients. J Bone Joint Surg Am..

[15] Reddy SC, Mann JA, Mann RA, Mangold DR (2011). Correction of moderate to severe coronal plane deformity with the STAR ankle prosthesis. Foot Ankle Int..

[16] Hobson SA, Karantana A, Dhar S (2009). Total ankle replacement in patients with significant pre-operative deformity of the hindfoot. J Bone Joint Surg Br..

[17] Joo SD, Lee KB (2017). Comparison of the outcome of total ankle arthroplasty for osteoarthritis with moderate and severe varus malalignment and that with neutral alignment. Bone Joint J..

[18] Demetracopoulos CA, Cody EA, Adams SB, DeOrio JK, Nunley JA, Easley ME (2019). Outcomes of total ankle arthroplasty in moderate and severe valgus deformity. Foot Ankle Spec..

[19] Gougoulias N, Khanna A, Maffulli N (2010). How successful are current ankle replacements?: a systematic review of the literature. Clin Orthop Relat Res..

[20] Gougoulias N, Maffulli N (2015). Osteotomies for managing varus and valgus malalignment with total ankle replacement. Clin Podiatr Med Surg..

[21] Lee KJ, Wang SH, Lee GW, Lee KB (2017). Accuracy assessment of measuring component position after total ankle arthroplasty using a conventional method. J Orthop Surg Res..

[22] Lee GW, Wang SH, Lee KB (2018). Comparison of intermediate to long-term outcomes of total ankle arthroplasty in ankles with preoperative varus, valgus, and neutral alignment. J Bone Joint Surg Am..

[23] Cody EA, Bejarano-Pineda L, Lachman JR (2019). Risk factors for failure of total ankle arthroplasty with a minimum five years of follow-up. Foot Ankle Int..

[24] Cody EA, Lachman JR, Gausden EB, Nunley JA, Easley ME (2019). Lower bone density on preoperative computed tomography predicts periprosthetic fracture risk in total ankle arthroplasty. Foot Ankle Int..

[25] Lavernia CJ, Alcerro JC, Contreras JS, Rossi MD (2011). Patient perceived outcomes after primary hip arthroplasty: does gender matter?. Clin Orthop Relat Res..

[26] Khashan M, Mor A, Beer Y (2014). Gait metric profile and gender differences in hip osteoarthritis patients. A case-controlled study. Hip Int..

[27] Towle KM, Monnot AD (2016). An assessment of gender-specific risk of implant revision after primary total hip arthroplasty: a systematic review and meta-analysis. J Arthroplasty..

[28] Cherian JJ, O'Connor MI, Robinson K, Jauregui JJ, Adleberg J, Mont MA (2015). A prospective, longitudinal study of outcomes following total knee arthroplasty stratified by gender. J Arthroplasty..

[29] Lim JB, Chi CH, Lo LE (2015). Gender difference in outcome after total knee replacement. J Orthop Surg (Hong Kong)..

[30] MacDonald SJ, Charron KD, Bourne RB, Naudie DD, McCalden RW, Rorabeck CH (2008). The John Insall Award: gender-specific total knee replacement: prospectively collected clinical outcomes. Clin Orthop Relat Res..

[31] Gen LY, Bin Abd Razak HR, Chi CH, Chye TH (2015). No gender-based differences in outcomes after conventional total knee arthroplasty in Asians. J Arthroplasty..

[32] Domsic RT, Saltzman CL (1998). Ankle osteoarthritis scale. Foot Ankle Int..

[33] Kitaoka HB, Alexander IJ, Adelaar RS, Nunley JA, Myerson MS, Sanders M (1994). Clinical rating systems for the ankle-hindfoot, midfoot, hallux, and lesser toes. Foot Ankle Int..

[34] Ware JE, Sherbourne CD (1992). The MOS 36-item short-form health survey (SF-36). I. Conceptual framework and item selection. Med Care..

[35] Coe MP, Sutherland JM, Penner MJ, Younger A, Wing KJ (2015). Minimal clinically important difference and the effect of clinical variables on the ankle osteoarthritis scale in surgically treated end-stage ankle arthritis. J Bone Joint Surg Am..

[36] Lee KB, Cho SG, Hur CI, Yoon TR (2008). Perioperative complications of HINTEGRA total ankle replacement: our initial 50 cases. Foot Ankle Int..

[37] Primadi A, Xu HX, Yoon TR, Ryu JH, Lee KB (2015). Neurologic injuries after primary total ankle arthroplasty: prevalence and effect on outcomes. J Foot Ankle Res..

[38] Quatman CE, Ford KR, Myer GD, Paterno MV, Hewett TE (2008). The effects of gender and pubertal status on generalized joint laxity in young athletes. J Sci Med Sport..

[39] Mohammad HR, Kennedy JA, Mellon SJ, Judge A, Dodd CA, Murray DW (2020). The clinical outcomes of cementless unicompartmental knee replacement in patients with reduced bone mineral density. J Orthop Surg Res..

[40] James SJ, Mirza SB, Culliford DJ, Taylor PA, Carr AJ, Arden NK (2014). Baseline bone mineral density and boneturnover in pre-operative hip and knee arthroplasty patients. Bone Joint Res..

[41] Ferber R, Davis IM, Williams DS (2003). Gender differences in lower extremity mechanics during running. Clin Biomech (Bristol, Avon).

[42] Landry SC, McKean KA, Hubley-Kozey CL, Stanish WD, Deluzio KJ (2007). Neuromuscular and lower limb biomechanical differences exist between male and female elite adolescent soccer players during an unanticipated run and crosscut maneuver. Am J Sports Med..

[43] Benyamini Y, Leventhal EA, Leventhal H (2000). Gender differences in processing information for making self-assessments of health. Psychosom Med..

[44] Morakis A, Tournis S, Papakitsou E, Donta I, Lyritis GP (2011). Decreased tibial bone strength in postmenopausal women with aseptic loosening of cemented femoral implants measured by peripheral quantitative computed tomography (pQCT). J Long Term Eff Med Implants..

[45] Runolfsdottir HL, Sigurdsson G, Franzson L, Indridason OS (2015). Gender comparison of factors associated with age-related differences in bone mineral density. Arch Osteoporos..

[46] Namba RS, Cafri G, Khatod M, Inacio MC, Brox TW, Paxton EW (2013). Risk factors for total knee arthroplasty aseptic revision. J Arthroplasty..

